# Decryption of the survival “black box”: gene family expansion promotes the encystment in ciliated protists

**DOI:** 10.1186/s12864-024-10207-3

**Published:** 2024-03-18

**Authors:** Didi Jin, Chao Li, Xiao Chen, Yurui Wang, Khaled A. S. Al-Rasheid, Naomi A. Stover, Chen Shao, Tengteng Zhang

**Affiliations:** 1 Laboratory of Biodiversity and Evolution of Protozoa in Wetland, College of Life Sciences, Shaanxi Normal University, Xi’an, 710119 China; 2 Key Laboratory of Evolution & Marine Biodiversity (Ministry of Education), and Institute of Evolution & Marine Biodiversity, Ocean University of China, Qingdao, 266003 China; 3 Laboratory of Marine Protozoan Biodiversity and Evolution, Marine College, Shandong University, Weihai, 264209 China; 4 Suzhou Research Institute, Shandong University, Suzhou, 215123 China; 5 Zoology Department, College of Science, King Saud University, Riyadh, 11451 Saudi Arabia; 6 Department of Biology, Bradley University, Peoria, 61625 USA

**Keywords:** Hypotrich, Pseudourostyla cristata, Encystment, Gene family expansion, Chitin synthase

## Abstract

**Background:**

Encystment is an important survival strategy extensively employed by microbial organisms to survive unfavorable conditions. Single-celled ciliated protists (ciliates) are popular model eukaryotes for studying encystment, whereby these cells degenerate their ciliary structures and develop cyst walls, then reverse the process under more favorable conditions. However, to date, the evolutionary basis and mechanism for encystment in ciliates is largely unknown. With the rapid development of high-throughput sequencing technologies, genome sequencing and comparative genomics of ciliates have become effective methods to provide insights into above questions.

**Results:**

Here, we profiled the MAC genome of *Pseudourostyla cristata*, a model hypotrich ciliate for encystment studies. Like other hypotrich MAC genomes, the *P. cristata* MAC genome is extremely fragmented with a single gene on most chromosomes, and encodes introns that are generally small and lack a conserved branch point for pre-mRNA splicing. Gene family expansion analyses indicate that multiple gene families involved in the encystment are expanded during the evolution of *P. cristata*. Furthermore, genomic comparisons with other five representative hypotrichs indicate that gene families of phosphorelay sensor kinase, which play a role in the two-component signal transduction system that is related to encystment, show significant expansion among all six hypotrichs. Additionally, cyst wall-related chitin synthase genes have experienced structural changes that increase them from single-exon to multi-exon genes during evolution. These genomic features potentially promote the encystment in hypotrichs and enhance their ability to survive in adverse environments during evolution.

**Conclusions:**

We systematically investigated the genomic structure of hypotrichs and key evolutionary phenomenon, gene family expansion, for encystment promotion in ciliates. In summary, our results provided insights into the evolutionary mechanism of encystment in ciliates.

**Supplementary Information:**

The online version contains supplementary material available at 10.1186/s12864-024-10207-3.

## Background

Encystment is a critical and ubiquitous survival strategy employed by microbial organisms to endure harsh environmental conditions [1–4]. As model organisms for many areas of research, single-celled ciliated protists (ciliates) are characterized by cilia and nuclear dimorphism (germline micronucleus [MIC] and somatic macronucleus [MAC] in one cell) [5–7]. Among them, many ciliate species undergo a typical lifestyle of encystment [8, 9] where they form resting cysts as an adaptive strategy against unfavorable conditions such as starvation, high population density, and salinity variation. However, although morphological and physiological data of encystment are available for approximately 40 species [1, 10], the evolution basis and mechanism of encystment in ciliates are poorly known, becoming a “black box” attracting extensive attention from biologists [3, 9, 11, 12].

Among ciliates, the subclass Hypotrichia represents an evolutionary pinnacle with complicated and specialized ciliary structures [8, 13, 14], all of which could degenerate during encystment and recover when excysting [4, 15], making them ideal organisms for studying encystment [1, 3, 16]. With the rapid development of high-throughput sequencing technologies, genomic sequencing and comparison analyses of ciliates have become effective methods to provide insights into above questions. However, despite the high number of morphospecies found in hypotrichs [8, 17], very little genomic data (publicly available for six species of hypotrichs) is available until now, primarily due to difficulties of bulk cultivation in laboratory conditions [18–22].

In this study, we achieved bulk cultivation and MAC genome sequencing of *Pseudourostyla cristata*, a model hypotrich ciliate for encystment studies. Following this sequencing, comparative genomic analyses of six representative hypotrichs were performed to further investigate their genomic features and to help elucidate the evolutionary basis of encystment. Our results revealed not only the genomic structures and diversity of hypotrichs, but also the impact of key evolutionary event, gene family expansion of genes involved in encystment. In addition, we also identified chitin synthase genes crucial for the encystment of ciliates and investigated their phylogenetic relationships, expression levels, gene structures, protein motifs, and conserved domains to further understand their evolution and biological functions.

## Results

### Assembly and annotation of the MAC genome of *Pseudourostyla cristata*

**Fig. 1:**
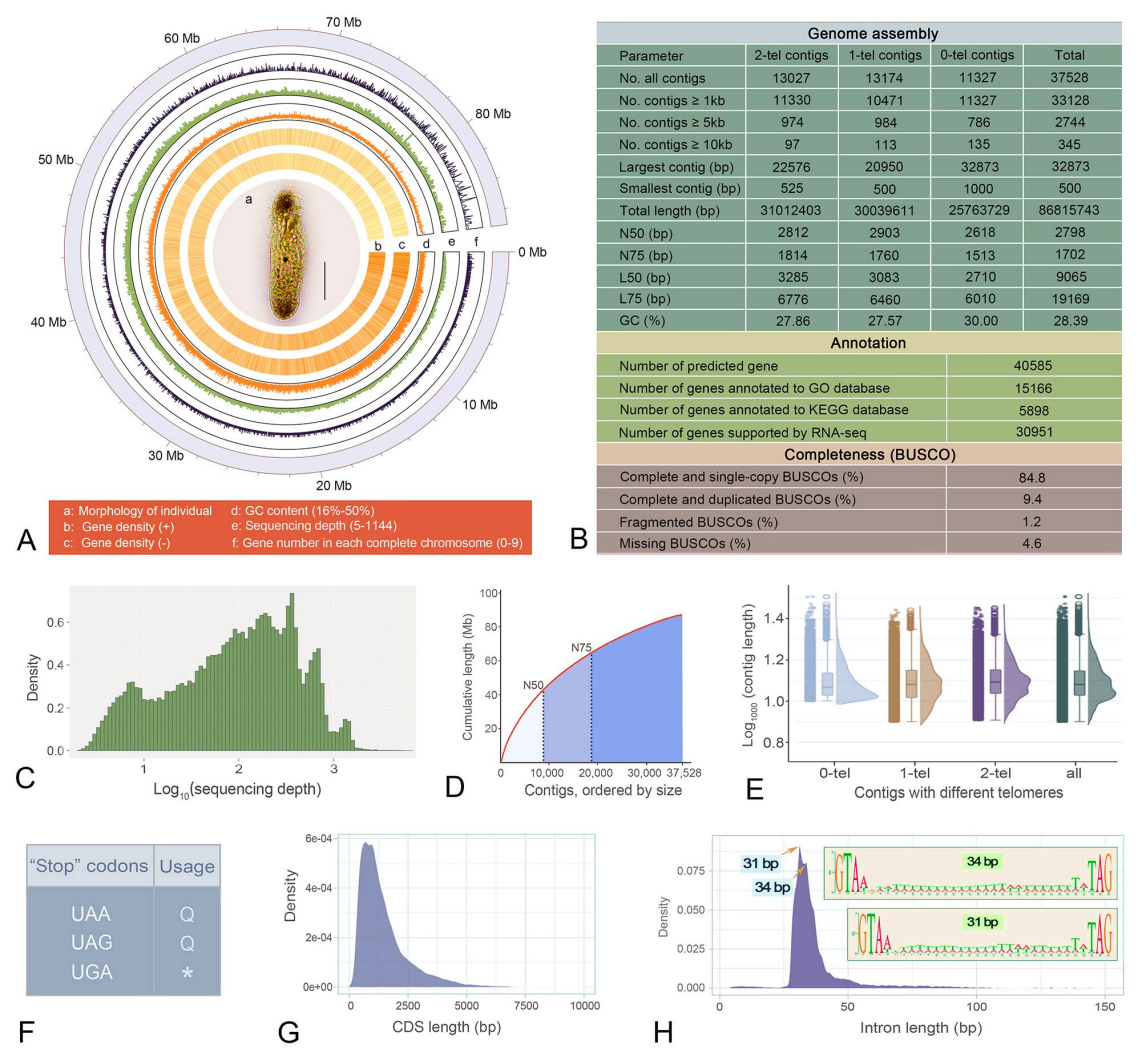
Sequencing, assembly, and features of the macronuclear genome of *Pseudourostyla cristata*. **A** Characteristics of all contigs and morphology of *P. cristata*. Ventral view in vivo is shown in a. Tracks b to f represent the distribution of gene density in sense strand (+), the distribution of gene density in antisense strand (-), GC content, genome coverage of reads, gene number of complete chromosomes, with densities calculated in 100-kb, 100-kb, 1-kb, 100-kb, 2-kb windows, respectively. All contigs are arranged from small to large in size (the outermost circle). **B** Statistics on assembly and annotation information of the macronuclear genome of *P. cristata*. Alveolate database (alveolata_odb10) was used for BUSCO analysis. **C** Distribution pattern of sequencing depth of all contigs. **D** The cumulative distribution of contig length. **E** The length distribution of contigs with different telomeres (0, 1 and 2). **F** The usage of three typical stop codons in *P. cristata* (Q, Glutamine; *, stop). **G** The length distribution of coding sequence (CDS). **H** The length distribution of intron sequence. Motifs of 31 and 34 bp introns are listed on the top right

**Fig. 2:**
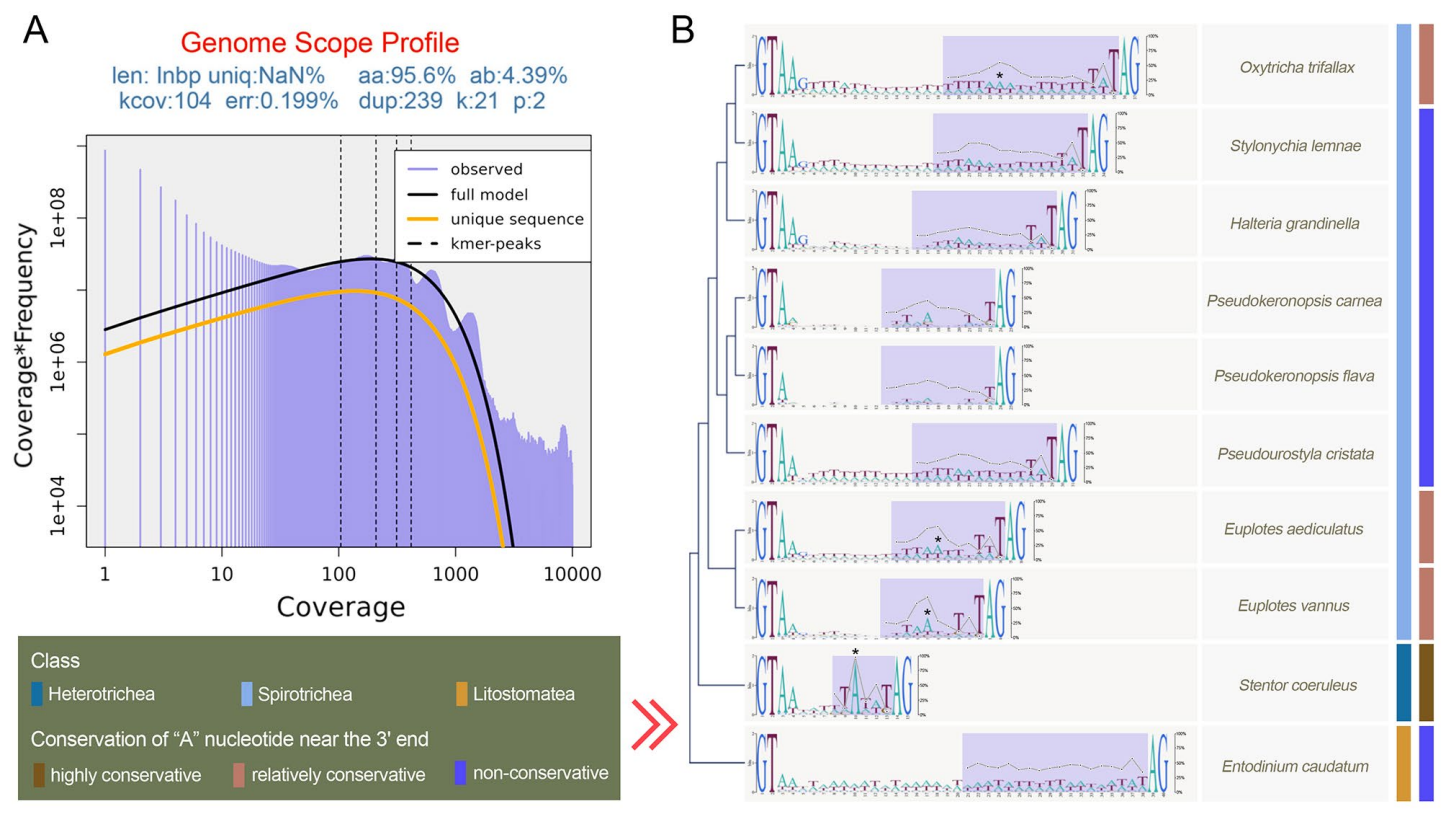
Analyses of heterozygosity of MAC genome (A), intron structure (B) in *Pseudourostyla cristata* and nine representative ciliates. **A** Log plot of a Kmer spectral genome composition analysis of *P. cristata.* Heterozygosity was estimated by jellyfish and GenomeScope2, based on 21-mers in Illumina sequence reads of the MAC genome. The len = inferred haploid genome length, uniq = percentage non-repetitive sequence, aa = Homozygous, ab = Heterozygous, kcov = mean kmer coverage for heterozygous bases, err = error rate of the reads, dup = average rate of read duplications, k = Kmer, p = ploid. The observed 21-mers frequency distribution is depicted in purple. The black lines represent the modeled distribution of 21-mers in the full genome. The orange lines represent the modeled distribution of the unique fraction of the genome. We find ~ 104× and ~ 208× coverage for heterozygous and homozygous peaks in our dataset, respectively. **B** Motif sequences of intron with most abundant size category in ten representative ciliates. The purple shadow and polyline show the percentage of base A at each position in the second half of intron sequences. Asterisks represent the conserved A nucleotide (The percentage of base A at the corresponding position is greater than 50%) which most likely represents a branch site

The MAC genome of *P. cristata* is 86.81 Mb in size and highly fragmented (Fig. 1A, B), similar to previously reported genomes of spirotrich ciliates [19, 20, 23, 24]. The genome assembly consists of 37,528 contigs (N50: 2,798 bp), with an average sequencing depth of 209.37× and an average GC content of 28.39% (Fig. 1B–D). Among these contigs, 34.7% (13,027) are canonical nanochromosomes with an average size of 2.4 kb and capped by repeated C4A4 and T4G4 telomeres (average size: 21 bp) at both ends (Fig. 1B, E). In addition, 35.1% of the contigs (13,174) are capped with one telomere, while no telomere sequences are found in the remaining 30.2% (11,327) contigs (Fig. 1B). We produced transcriptome information to validate the quality of the *P. cristata* genome assembly and mapped the RNA-seq and DNA-seq reads to the genome assembly, with mapping ratios of 94.75% and 92.65%, respectively. Genome assembly completeness was also evaluated using BUSCO, which showed that 94.2% of complete orthologs can be detected in the Alveolata database (Fig. 1B). Additionally, Kmer distribution (Fig. 2A) indicates that the *P. cristata* genome has high heterozygosity (4.39%).

Stop codon usage analysis revealed a reassignment phenomenon in *P. cristata* (Fig. 1F). Specifically, UAA and UAG encode glutamine, with only UGA used as a stop codon. The MAC genome was annotated based on stop codon reassignment, and a total of 40,585 genes were identified. Among these, 37.4% (15,166) and 14.5% (5,898) of the genes had hits in the Gene Ontology (GO) and Kyoto Encyclopedia of Genes and Genomes (KEGG) databases, respectively (Fig. 1B). The vast majority of the canonical nanochromosomes (82.3%) contain only a single gene, while 13.7% contain two genes and 4.0% contain three or more genes (Fig. 1A). The average lengths of gene and coding sequence (CDS) regions are 1,627 bp and 1,535 bp, respectively (Fig. 1G). Approximately half of *P. cristata* genes lack introns. When introns are present, most (72.43%) range from 28 to 40 bp, with two peaks at 31 and 34 bp, showing a canonical GT–AG motif (Figs. 1H and 2B).

To understand which genomic features are related to nanochromosome length, we performed Spearman correlation analysis between nanochromosome length and different genomic features, including gene number and length, subtelomeric and intergenic region length, GC content, telomere length, CDS, and intron length. The results show that chromosomal length is most closely related to gene (Spearman’s *r* = 0.98) and CDS length (Spearman’s *r* = 0.97) (Fig. S1A). In addition, our analyses show that the CDS that makes up the majority of each nanochromosome largely determines its overall GC content (Fig. S1B).

### Genomic characteristics and orthogroups in Hypotrichia

**Fig. 3:**
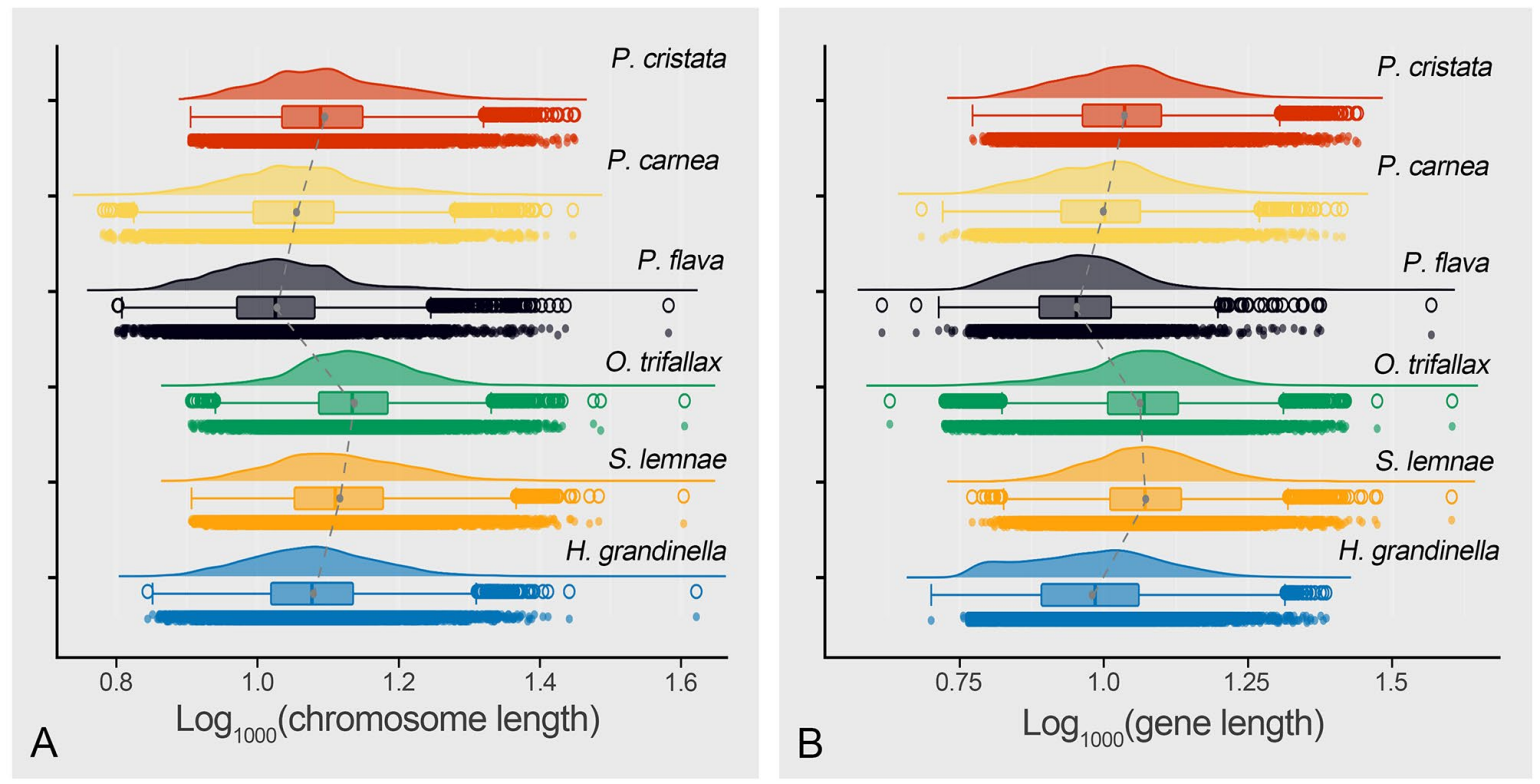
Comparison of chromosomes and genes length of MAC genome in six hypotrichous ciliates. **A** Comparison of the distribution of chromosome length. **B** Comparison of the distribution of gene length. The gray solid dots represent the average length of chromosomes in each species

**Fig. 4:**
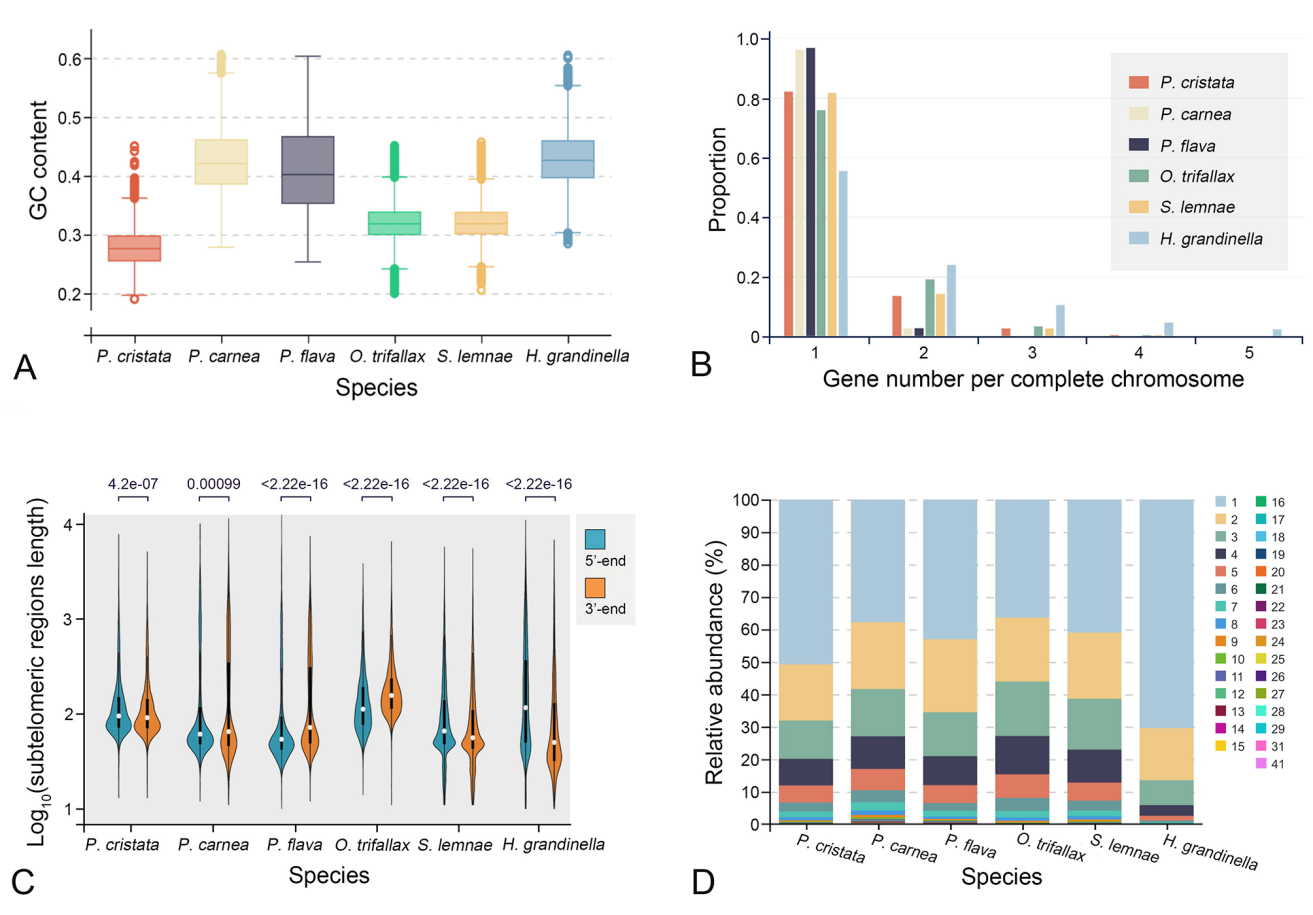
Characteristics of MAC genomes among six hypotrichous ciliates. **A–D** Comparison of the distribution of GC content, gene number per complete chromosome, subtelomeric regions length, and exon number per gene. The numbers in (C) represent the *p*-value. The number in the legend of (D) represents the exon number per gene

Generally, MAC genomes of hypotrichous ciliates are fragmented into gene-sized nanochromosomes [21, 22, 25]. To further dissect the genomic features of hypotrichs, we identified and compared complete chromosomes containing telomeres at both ends in six hypotrichous ciliate genomes (*Oxytricha trifallax*, *Stylonychia lemnae*, *Halteria grandinella*, *Pseudourostyla cristata, Pseudokeronopsis flava*, and *Pseudokeronopsis carnea*) (Figs. 3 and 4). Among these six species, the genome of *P. cristata* has the lowest GC content (< 30%) (Fig. 4A; Table S1). *P. carnea* and *P. flava* have higher proportions of single-gene chromosomes (96.6% on average) than other species, and the proportion of single-gene chromosomes in *H. grandinella* is the lowest (56%) (Fig. 4B). The length distribution of the subtelomeric regions of the nanochromosomes also shows interspecific differences. The 5ʹ subtelomeric regions upstream of the gene in *P. cristata*, *S. lemnae*, and *H. grandinella* tend to be longer than that of the 3ʹ end, whereas the opposite is observed in *P. carnea*, *P. flava*, and *O. trifallax* (Fig. 4C). In addition, *P. cristata* and *H. grandinella* have a higher proportion of single-exon genes (50.8% and 70.4%, respectively) than other species (39.6% on average) (Fig. 4D).

**Fig. 5:**
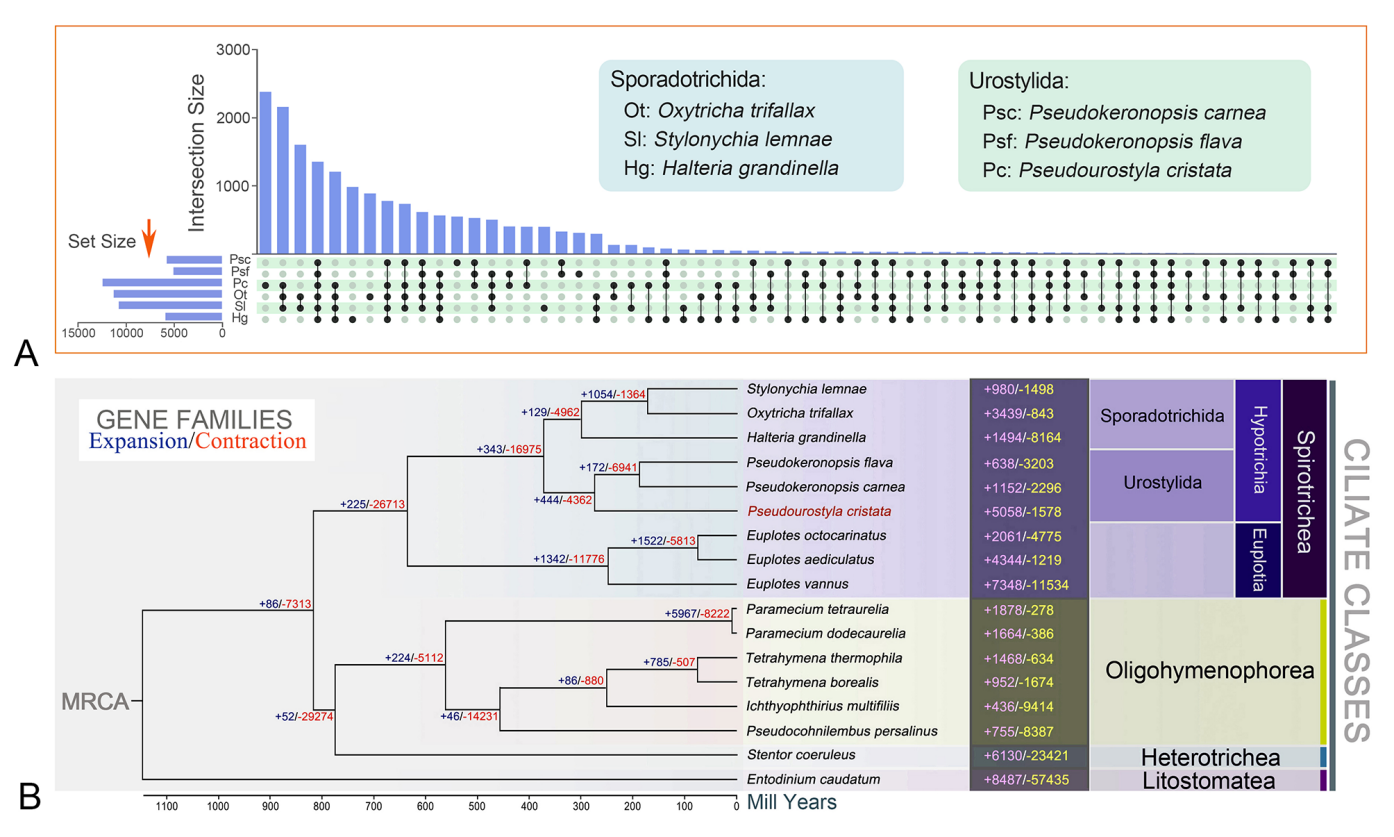
Analyses of orthogroups and gene family expansion/contraction within ciliates. **A** Upset plot of intersecting sets of orthogroups in six hypotrichous ciliates. **B** Phylogenomic tree with divergence time, and gene family expansion/contraction for *Pseudourostyla cristata* and 16 other species. The numbers at nodes indicate the number of expanded (blue) and contracted (orange) gene families at different evolutionary time points. Numbers following species names represent expanded and contracted gene families for species alone. MRCA, most recent common ancestor

In the present study, orthogroups were determined in *P. cristata* and the other five hypotrichous ciliates. A total of 1,349 orthogroups are shared in all six MAC genomes (Fig. 5A). Among them, the three species belonging to the order Urostylida (*P. cristata*, *P. carnea*, and *P. flava*) share 524 orthogroups (Fig. 5A), whereas the other three species of the order Sporadotrichida (*O. trifallax*, *S. lemnae*, and *H. grandinella*) share only 292 orthogroups (Fig. 5A).

### Gene family expansion

Gene family expansion is widespread during the genomic evolution of nearly all major phyla of eukaryotes, including ciliates [22, 23, 26]. Furthermore, it is often associated with the adaptation of organisms to variable environments [27, 28]. To investigate the effect of gene family expansion on encystment, this phenomenon in representative hypotrichs was identified and analyzed here.

**Fig. 6:**
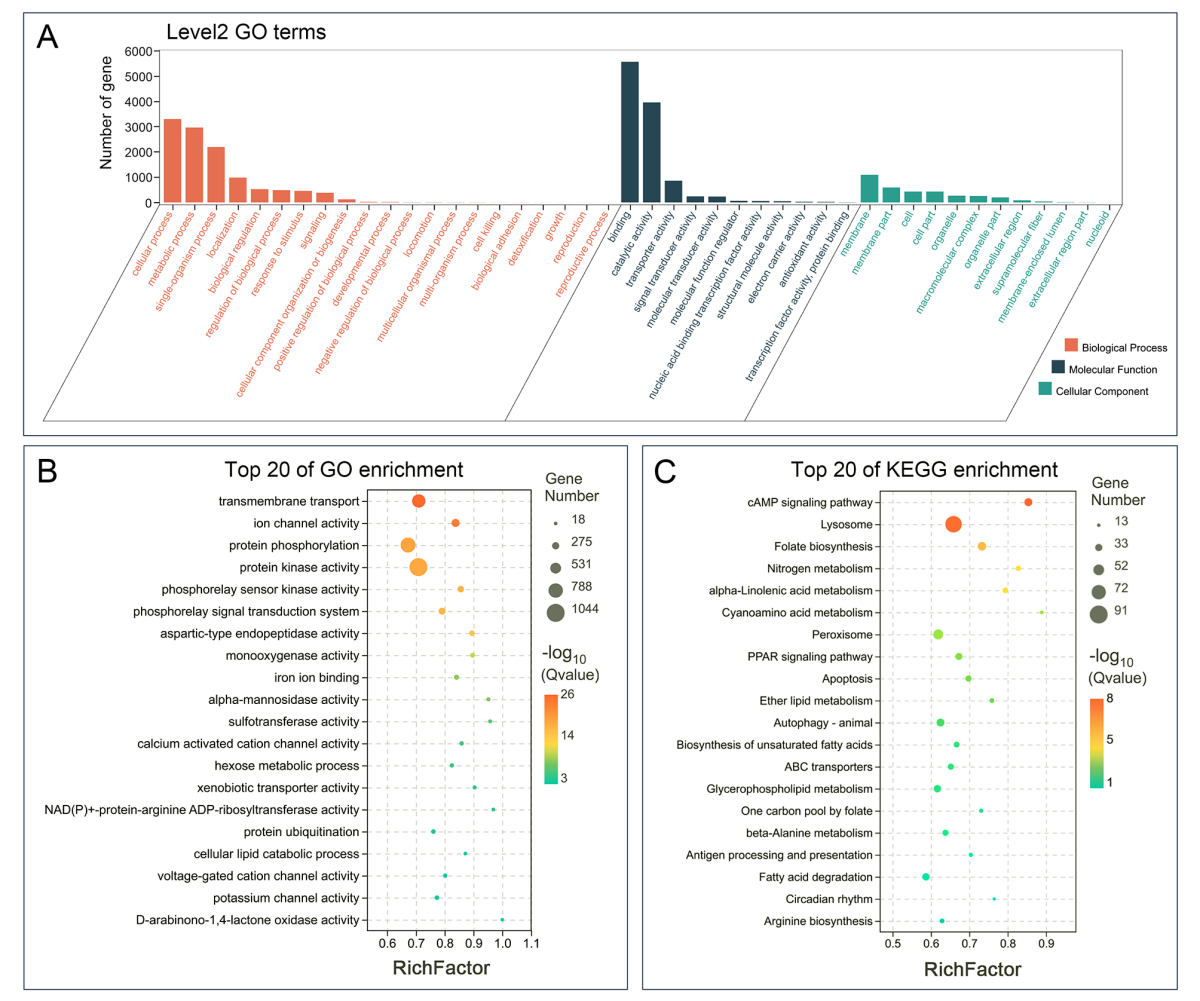
GO and KEGG pathway enrichment analyses of significantly expanded gene families. **A** Level 2 GO terms associated with significantly expanded gene families in *Pseudourostyla cristata*. **B, C** GO (B) and KEGG (C) enrichment bubble plots of *Pseudourostyla cristata*. The top 20 GO terms and KEGG pathways with the smallest Q-values are shown, with the ordinate as the GO term or KEGG pathway and the abscissa as the RichFactor. GO, Gene Ontology; KEGG, Kyoto Encyclopedia of Genes and Genomes. RichFactor = the ratio of enriched gene number to all gene number in this pathway term

A high-confidence phylogenetic tree and an estimated divergence time (involving Spirotrichea, Oligohymenophorea, Heterotrichea, and Litostomatea) were constructed. Among spirotrichs, the greatest gain/loss of orthologs happened in *Euplotes vannus*. Furthermore, *P. cristata* also showed a large gene family expansion (5058 genes) (Fig. 5B). We then performed GO functional enrichment analyses for the expanded gene families of *P. cristata* (Fig. 6A), and the top-ranked GO terms were selected according to Q-values (Fig. 6B; only lower-level terms are shown to avoid redundancy). Among the enriched GO terms, ion channel activity, calcium-activated cation channel activity, voltage-gated cation channel activity, and potassium channel activity are associated with transmembrane transport. In addition, expanded gene families are also enriched for protein phosphorylation (catalyzed by protein kinases) and phosphorelay signal transduction systems, which involve autophosphorylation of histidine kinases (Fig. 6B). Similarly, KEGG functional enrichment analyses were performed for the expanded gene families, and the top 20 enriched KEGG pathways are shown in Fig. 6C. These results indicate that the top two pathways are the cAMP signaling pathway and lysosome functions. Genes involved in apoptosis and autophagy are also significantly enriched (Fig. 6C).

Additionally, the GO enrichment analyses on expanded gene families of the other five hypotrichs were conducted. The results show that phosphorelay sensor kinase activity (synonym: two-component system sensor activity) is enriched for all six hypotrichs (Fig. S2).

### Analysis of chitin synthase gene/protein sequences for nine representative species

Chitin synthases (CHS) are membrane-inserted glycosyltransferases with multiple transmembrane domains that are found extensively in eukaryotes (e.g., fungi, insects, crustaceans, algae, and protists) [29, 30]. Yang & Fukamizo (2019) [31] suggested that the presence or absence of *CHS* genes can be considered a marker for chitin (a β-1-4-linked *N*-acetylglucosamine polymer) biosynthesis. In ciliates, chitin has been detected in the lorica, a shell-like protective outer covering of heterotrichs (e.g., *Folliculinopsis producta*) and in the cyst walls of other taxa (e.g., *Oxytricha fallax* and *Tetrahymena rostrata*) [31, 32]. Chitin is important for the encystment of ciliates as it can protect cysts against physical/chemical environmental stresses and support the shape of the cyst [32].

**Fig. 7:**
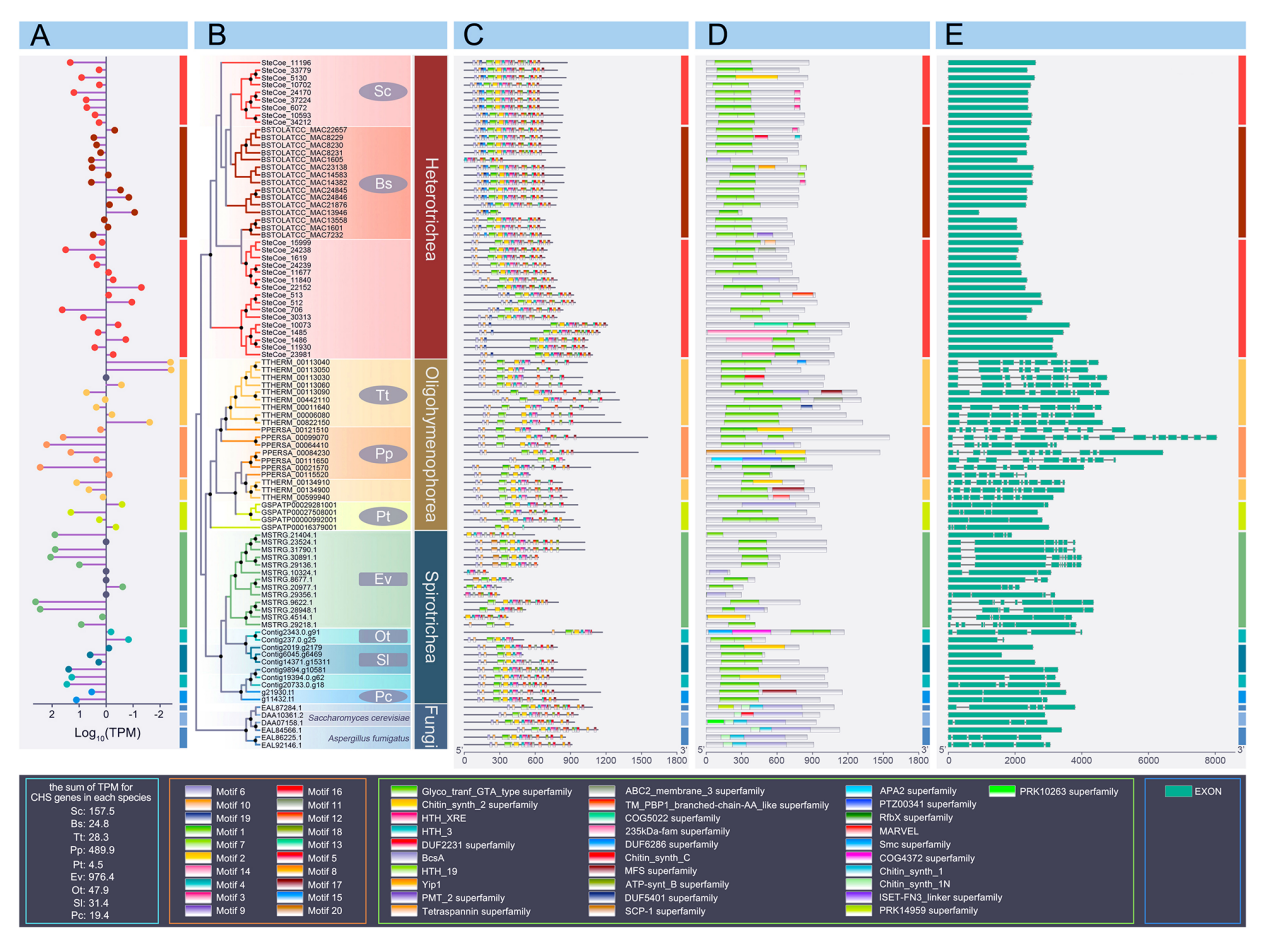
Consensus tree based on eighty-six chitin synthases (CHS) proteins from nine ciliates and six CHS proteins from two fungi, with corresponding information of gene expression, motif, conserved domain, and gene structure. **A** Normalized gene expression level of different *CHS* genes. Data are represented as log10-transformed ratios of TPM values [log10(TPM value)]. Grey dots at the vertical axis indicate corresponding genes not supported by RNA-seq reads. **B** Consensus tree inferred from 86 CHS proteins of nine ciliates and six CHS proteins of two fungi. Sc, *Stentor coeruleus*; Bs, *Blepharisma stoltei*; Ev, *Euplotes vannus*; Sl, *Stylonychia lemnae*; Ot, *Oxytricha trifallax*; Pc, *Pseudourostyla cristata*; Tt, *Tetrahymena thermophila*; Pp, *Pseudocohnilembus persalinus*; Pt, *Paramecium tetraurelia*. Elliptical and rectangular profiles represent that the pellicle of ciliates is soft and rigid, respectively. Black circles indicate that node support values greater than 95% (high confidence value). **C** MEME motif distribution of each protein. **D** Conserved domain of each protein. **E** Corresponding gene structure of each CHS protein. The green boxes in (E) represent exons, lines represent introns. The four boxes on the black background below the figure are the sum of TPM for *CHS* genes in each species and the legends of (C), (D), and (E), respectively

In this study, to better understand the diversification and evolution of CHS genes/proteins in ciliates, phylogenetic and structural analyses were performed using 86 CHS protein sequences from nine representative ciliates of three classes and six CHS protein sequences from two fungi (Fig. 7). The expression and models for most of these putative *CHS* genes are supported by RNA-seq data (Fig. 7A). In addition, we mapped the genetic profiles, including gene expression, conserved motifs, protein domains, and exon organization of CHS proteins onto a phylogenetic tree (Fig. 7B–E).

In our consensus tree, all ciliate CHS proteins are divided into three groups corresponding to the classes Heterotrichea, Oligohymenophorea, and Spirotrichea (Fig. 7B). Interestingly, only the CHS proteins of *E. vannus* form a monophyletic clade, whereas other ciliates form paraphyletic clades (Fig. 7B). The relative abundance of *CHS* gene expression was determined by calculating the transcripts per million (TPM) values (Fig. 7A). The sum of the TPM for *CHS* genes in *Euplotes vannus* is the highest among these nine species (Fig. 7A). The *S. coeruleus* and *P. cristata* have the largest number of CHS protein homologs (25), and the fewest of these proteins (2), respectively (Fig. 7B). Interestingly, both of these species have soft pellicles. There are 13, 4, and 4 CHS proteins in *E. vannus*, *Stylonychia lemnae*, and *Oxytricha trifallax* with rigid pellicles, respectively (Fig. 7B).

For CHS protein motifs in ciliates (Fig. S3), motifs 1–7 and 10 are detected in most CHS proteins (≥ 80%), and CHS proteins of *S. coeruleus* generally have more motifs than those of other species (each CHS protein of *S. coeruleus* has at least 75% of all motifs, while others possess at least 30–50%) (Fig. 7C). Notably, motifs 14 and 18 are class-specific in Heterotrichea, whereas motifs 19 and 20 exist only in the CHS proteins of *E. vannus* (Fig. 7C). For conserved domains, the Glyco_tranf_GTA_type superfamily exists in almost all ciliate CHS proteins (98.8%), corresponding to the positions of motifs 6, 10, 19, 1, 7, 2, 14, 4, 3, and 9 (Fig. 7D). Additionally, most *CHS* genes with close evolutionary relationships have approximately the same number of exons and similar exon positions (Fig. 7E). As an exception, all *CHS* genes in Heterotrichea have only one exon, whereas the *CHS* genes of the Oligohymenophorea and Spirotrichea species are mostly multi-exon genes (Fig. 7E).

## Discussion

### Features of the MAC genome and nanochromosomes in subclass Hypotrichia

The subclass Hypotrichia is one of the most morphologically diverse groups of ciliates [8, 17, 33, 34]. According to Lynn (2008) [8], they can be divided into three orders: Stichotrichida, Urostylida, and Sporadotrichida. This study reports the MAC genome of the urostylid ciliate *Pseudourostyla cristata*, which is highly fragmented into nanochromosomes. This is consistent with previous reports on the MAC genomes of other hypotrich ciliates [18–22, 25]. In addition, based on correlation analyses, we find that the length and GC content of the nanochromosomes are mainly determined by the coding region of the genes they carry (Fig. S1A, B). Furthermore, the comparison results of nanochromosomes also indicate high chromosomal structural variability based on the divergence of the subtelomeric region length, the proportion of single-gene nanochromosomes, and the proportion of single-exon genes.

In the present study, the average GC content of the MAC genomes in *Pseudokeronopsis flava*, *Pseudokeronopsis carnea*, and *Halteria grandinella* are higher than that in *P. cristata*, *Stylonychia lemnae*, and *Oxytricha trifallax* (Fig. 4A; Table S1). Unfortunately, this finding does not immediately suggest a link between GC content and lifestyles or cell phenotypes among these six hypotrichs. Previous studies have shown that the genomic GC content of anaerobic prokaryotes is significantly lower than that of aerobic prokaryotes [35]. Based on our review of existing research, we have found the GC contents of available MAC genomes in anaerobic rumen ciliates are generally less than 25% [36, 37], while average GC contents of aerobic ciliates are about 33.9% [21, 38]. However, there are also a few aerobic species (e.g., *Tetrahymena thermophila* [22.3%] and *Ichthyophthirius multifiliis* [15.9%]) have lower GC content than the anaerobic rumen ciliate, *Entodinium caudatum* (23%) [37, 39, 40]. Therefore, based on existing results, the differences in the GC content of ciliates cannot be explained simply by differences in oxygen demand, lifestyles, or cell phenotypes.

### Gene family expansion during the evolution of hypotrichs

Previous reports have indicated that an increased concentration of intracellular Ca^2+^ in ciliates may activate cyst formation through intracellular signaling pathways, such as the cAMP signaling pathway [9, 41]. More specifically, an increase in Ca^2+^ leads to a higher cAMP concentration, which then in turn activates the phosphorylation of proteins involved in encystment induction by cAMP-dependent kinase [12, 41].

In the present study, we have identified a set of expanded gene families in *P. cristata*, many of which show an enrichment in GO terms and KEGG pathways related to encystment induction (Fig. 6B, C). The GO terms “ion channel activity” and “protein phosphorylation” are significantly enriched, indicating that active transport of Ca^2+^ and phosphorylation of encystment-related proteins may be enhanced in *P. cristata* (Fig. 6B). Similarly, the cAMP signaling pathway is enriched in the KEGG pathway analyses (Fig. 6C). According to previous studies, rapid changes in cell structure and morphology during ciliate encystment are usually accompanied by protein degradation involving two organelles, lysosomes and peroxisomes [42–44]. Coincidentally, both related pathways are enriched based on the KEGG analyses (Fig. 6C). In addition, the encystment process may involve apoptotic and autophagic pathways [43, 45], which are also detected in this study (Fig. 6C). These results suggest that induction of encystment genes is enhanced in the MAC genome of *P. cristata*, which helps *P. cristata* respond better and faster to external stimuli and adversity. This feature is consistent with years of previous research on this ciliate model for encystment, which was chosen specifically due to the ease with which encystment can be induced [9, 43, 46, 47]. Additionally, based on our previous research, gene families associated with encystment (chitin metabolism and FoxO signaling pathway) also expanded in another spirotrich species *Euplotes aediculatus* [23]. All of these two findings suggest that the gene family expansion related to encystment in *P. cristata* may not be an isolated case, as other ciliates also tend to expand gene families involved in encystment during evolution to enhance survivability.

GO enrichment results of expanded gene families in six hypotrichs highlight the importance of genes related to phosphorelay sensor kinases in hypotrichs (Fig. S2). Phosphorelay sensor kinases belong to two-component signal transduction systems, which are the principal means of coordinating responses to environmental changes in prokaryotes, plants, fungi, and protozoa [48]. In ciliates, the activation of encystment and excystment is closely linked to the activation of signal transduction pathways [4, 9, 45]. Our findings here demonstrate that hypotrichs have enhanced their signal transduction capabilities by enrichment of phosphorelay sensor kinases, allowing them to trigger the encystment in a wide range of adverse/suitable environments.

### Chitin synthase

Previous studies have shown that chitin is the main component of the ciliate cyst walls. Unfortunately, little effort has been made to determine the distribution, diversity, and evolution of chitin synthase (*CHS*) genes, which are key genes for chitin production among ciliates. In this study, a phylogenetic tree based on all available homologous CHS proteins of nine ciliates and two fungi was constructed (Fig. 7B), and shows that *CHS* genes are found in all three classes (Spirotrichea, Oligohymenophorea, Heterotrichea). Previous studies have indicated the presence of chitin in other taxa, such as *Bursaria truncatella* in Colpodea and *Nassulopsis lagenula* in Nassophorea [32]. Consequently, we believe that *CHS* genes may be ancient genes in ciliates. In our tree, the CHS proteins are paraphyletic in eight species (except for *Euplotes vannus*), illustrating that CHSs have a complex evolutionary history in ciliates. The RNA-seq data of the normal cell form (non-cyst) are all available for these nine ciliates, and gene expression analyses were performed based on these data, showing that *CHS* genes can be expressed in non-cyst ciliates (Fig. 7A). Considering the function of chitin, we suspect that chitin produced in the normal cell stage may be related to the pellicle of ciliates. However, in the present study, the number of CHS proteins in ciliates with rigid pellicles (*Oxytricha trifallax*, *Stylonychia lemnae*, and *E. vannus*) is not generally more than that in ciliates with soft pellicles (*Stentor coeruleus*, *Blepharisma stoltei*, *Tetrahymena thermophila*, *Pseudocohnilembus persalinus*, *Paramecium tetraurelia*, and *Pseudourostyla cristata*) (Fig. 7B). Therefore, one possibility is that chitin is useful for pellicles but may not play a major role in the rigid pellicle. However, more evidence and information are needed to clarify the function of chitin in vegetative cells.

Domain analyses of CHS proteins show that the Glyco_tranf_GTA_type superfamily (glycosyltransferase family A) is the most highly conserved domain shared by almost all CHS proteins of ciliates, strongly supporting our prediction of *CHS* homologs (as mentioned above, CHSs are glycosyltransferases) (Fig. 7D). In addition, the motifs of CHS proteins show that motifs 1–7 and 10 exist in almost all CHS proteins, which is generally consistent with the position of the above domain in the Glyco_tranf_GTA_type superfamily, indicating that these motifs are hallmarks of the Glyco_tranf_GTA_type superfamily (Fig. 7C). However, some CHS proteins (e.g., BSTOLATCC_MAC1605 and MSTRG.29356.1) also lack these motifs and the corresponding domains (Fig. 7C). This may be due to incomplete gene assembly or annotation.

The exon structures of *CHS* genes in ciliates are species-specific; all *CHS* genes in Heterotrichea have only a single exon, whereas most *CHS* genes in Spirotrichea and Oligohymenophorea possess multiple exons (except for *S. lemnae* and *T. thermophila*) (Fig. 7E). According to phylogenetic research on ciliates, Heterotrichea usually occupies a basal position among the above three classes and has a much closer relationship with the most ancestral ciliated groups [8, 49]. Therefore, it is reasonable to assume that the one-exon *CHS* gene represents primitive and ancient characteristics. Introns might have been gradually acquired during evolution, resulting in multiple-exon genes. Coincidentally, a similar phenomenon is observed for all genes when examining the proportion of multi-exon genes in these nine species (Table 1). The results show that the proportion of multi-exon genes in Heterotrichea (18–20%) is significantly lower than that in Oligohymenophorea (70–91%) and Spirotrichea (48–66%), further supporting our hypothesis on the gene evolution trend from single-exon to multi-exon by acquiring introns (Table 1).

**Table 1:** The ratio of multi-exon genes in nine species among three class

Class	Species	The number of multi-exon genes	The number of all genes	The ratio of multi-exon genes
Heterotrichea	*Stentor coeruleus*	6218	30,478	0.20
	*Blepharisma stoltei*	4670	25,785	0.18
Oligohymenophorea	*Tetrahymena thermophila*	18,732	26,742	0.70
	*Pseudocohnilembus persalinus*	12,034	13,179	0.91
	*Paramecium tetraurelia*	31,615	39,580	0.80
Spirotrichea	*Euplotes vannus*	21,713	32,779	0.66
	*Oxytricha trifallax*	15,822	24,885	0.64
	*Stylonychia lemnae*	9980	15,228	0.66
	*Pseudourostyla cristata*	19,567	40,592	0.48

### Intron branch point in ciliates

Intron features of *Pseudourostyla cristata* are consistent with that of other ciliates [24, 50, 51]. According to previous studies, both the introns of *Euplotes vannus* (Spirotrichea) and *Stentor coeruleus* (Heterotrichea) exhibit a conserved “A” nucleotide near the 3ʹ end [50, 51], while such a conserved “A” nucleotide site is not found in intron sequences of *P. cristata* (Fig. 1H). We further analyzed the intron sequences with the most abundant size for representatives of Spirotrichea, Heterotrichea, and Litostomatea (Fig. 2B). The results show that a relatively conserved “A” nucleotide near the 3ʹ end is also found in *Euplotes aediculatus* and *Oxytricha trifallax*, similar to *E. vannus* and *S. coeruleus*, but is not detected in other species.

Slabodnick et al. (2017) suggested that the conserved “A” nucleotide near the 3ʹ end of intron sequences of *S. coeruleus* could represent a branch point [51]. The branch point is an internal intronic sequence that initiates a splicing event through hydrophilic attack by an adenosine 2ʹ hydroxyl group at the 5ʹ splice site during the removal of intron regions from the pre-messenger RNA [52, 53]. In addition, the sequence conservation of branch points varies among species, from highly conserved sequences in hemiascomycetous yeasts (e.g., *Saccharomyces cerevisiae*) and protists (e.g., *Trichomonas vaginalis* and *Giardia lamblia*) to highly divergent sequences (e.g., the nucleomorph of *Bigelowiella natans*) [52, 54, 55]. Similarly, our analyses indicate that the conservation of branch points varies across ciliates (Fig. 2B).

Pre-mRNA splicing is a crucial process in eukaryotes and is catalyzed by a spliceosome complex comprising five small nuclear ribonucleoproteins (snRNPs) and multiple non-snRNP-associated proteins [53, 56, 57]. Each snRNP comprises one or two snRNAs, a set of common Sm proteins, and a variable number of particle-specific proteins [53, 58]. Recently, Nuadthaisong et al. (2022) identified the presence of all snRNAs and conserved protein components of the spliceosome in *Stentor*, suggesting that conserved pre-mRNA splicing processes and mechanisms in eukaryotes exist in *Stentor* [56]. Similarly, in the present study, we found the presence of all snRNAs and most protein components of the spliceosome in *P. cristata* and *O. trifallax*. Therefore, we speculate that the mechanisms of pre-mRNA splicing are conserved in most ciliates. Nevertheless, it is worth noting that the average intron size in ciliates is usually smaller (median intron length: 15 bp [*S. coeruleus*] to 72 bp [*O. trifallax*]) (Fig. 2B) than that of other eukaryotes (median intron length: 98 bp [*Arabidopsis thaliana*] to 1,334 bp [humans]) [59]. Given that small-sized introns may cause steric clashes during pre-mRNA splicing [53, 56], it is supposed that the splicing mechanisms in ciliates may show some divergence from those with larger introns, although the splicing mechanism should be largely conserved.

## Conclusion

In the present study, we conducted comparative genomic analyses of hypotrich ciliates, the typical group with encystment lifestyle, and reported a newly sequenced MAC genome for one of its representative species, *Pseudourostyla cristata*. Our analyses revealed that gene families related to encystment induction were expanded in *P. cristata*. Among the six hypotrichs studied, there is a significant expansion of phosphorelay sensor kinase-related gene families belonging to the two-component signal transduction system believed to trigger encystment. Furthermore, the chitin synthase genes responsible for producing ciliate cyst walls were analyzed and compared, showing an evolutionary trend from a single exon structure to multiple exons. Additionally, the structures of MAC genomes and nanochromosomes were also characterized and compared in Hypotrichia. In summary, our study provides insights into the key evolutionary event and basis for encystment promotion in hypotrich ciliates, and greatly enriches the understanding on the genomic evolution of ciliates.

## Materials and methods

### Cell culture, DNA and RNA extraction, and Illumina sequencing

*Pseudourostyla cristata* cells were separated from a freshwater pond in Taipingjiao Park (120°22′2.12″, 36°3′31.7″) in Qingdao, China. Species was identified through morphological features and the SSU-rRNA gene. The SSU-rRNA gene sequence was deposited into the NCBI Sequence Read Archive with the accession number PP132854. Ten cells were collected, washed with distilled water, and incubated in cell culture flasks with 1% lettuce juice medium and *Klebsiella pneumoniae* as food resource at 25°C until reaching ∼200 cells/mL^− 1^. Cell harvest was performed by centrifugation at 200 g for 3 min. Genomic DNA was extracted using the phenol/chloroform/isoamyl alcohol method. Total RNA was extracted using the TRIzol Reagent (Invitrogen).

One DNA library and one RNA library were constructed with NEBNext Ultra DNA Library Prep Kit for Illumina (NEB, #E7370L, USA) and NEBNext Ultra RNA library prep kit for Illumina (NEB, #E7530S, USA), respectively. Sequencing was performed on an Illumina Hiseq2500 platform with paired-end 150 bp read length at Novogene (Novogene, Beijing, China).

### Genome assembly

All reads data were trimmed to remove adaptors with fastp v.0.23.1 (-q 20 -u 40 -l 36) [60], and only filtered paired-end reads (56.70 Gb) were retained for assembly. Basic features of the MAC genome such as heterozygosity and duplication rate were estimated by Jellyfish version 2.2.3 (parameters: -C -k 21) [61] and GenomeScope2 verison 2.0 (parameters: Kmer length = 21, Read length = 150) [62], based on 21-mers in primary sequence reads. *De novo* genome assembly was performed using MEGAHIT version 1.2.9 [63] and Spades version 3.14 [64], respectively. The two assembly drafts were merged by quickmerge version 0.3 [65], producing the primary contigs. These contigs were further merged by Cap3 (version date: 02/10/15) [66]. The putative contaminant (bacteria [mainly *Klebsiella pneumoniae*] and mitochondria) sequences were identified and removed using BLASTN verison 2.10.1+ (E-value cutoff = 1e-5) by searching against the mitochondrial genome of *Pseudourostyla cristata* and the vast majority of bacteria genome downloaded from GenBank [67]. The low-quality contigs (GC > 50% or coverage < 5×), amounting to a total of 1382, were also filtered and discarded. CD-HIT [68] was employed to identify and remove redundant contigs (similarity ≥ 95%), obtaining the final assembly result. The quality of the genome assembly was evaluated using QUAST version 5.0.2 [69].

### Detection of telomeres

According to previous studies, the telomere sequences of Spirotrichea are pretty conserved and are composed of repeated C4A4 and T4G4 sequences [37, 70]. In this work, these repeated C4A4 and T4G4 sequences were also detected at both ends of many contigs in *P. cristata*, suggesting that these repeated sequences serve as telomeres of *P. cristata*.

### Gene prediction and annotation

Codetta (https://github.com/Swart-lab/codetta) was used to predict the genetic code (codon table) of genome sequence in *Pseudourostyla cristata*. The *de novo* gene prediction was performed using AUGUSTUS version 3.4.0 [71] with hints of RNA-seq data and information of stop codon usage. The specific steps are as follows: (1) RNA-seq reads of *P. cristata* were mapped to the MAC genome assembly by HISAT2 version 2.1.0 [72], and the transcripts were assembled by StringTie version 1.3.7 [73]; (2) the assembled transcriptome was analyzed with TransDecoder version 5.5.0 (https://github.com/TransDecoder/TransDecoder), obtaining a relatively reliable genome annotation file (.gff3 format); (3) This annotation file was further transformed into a GeneBank (gb) file format for AUGUSTUS model training; (4) stop codon usage of *P. cristata* was used in the parameter file of AUGUSTUS model training. The completeness of the predicted gene set was analyzed using BUSCO version 5.2.2 [74] with Alveolata databases (alveolata_odb10). The sequences of intron and coding sequence (CDS) region were extracted using the TBtools software [75]. Then motifs of extracted sequences were searched using MEME [76].

Predicted genes were functionally characterized with InterProScan 5.52-86.0 [77] with options -goterms and -pa. GO [78] and KEGG [79] annotations were deduced for each gene based on the InterPro entries and KofamScan [80] results.

### Gene family analysis and divergence time estimation

Ciliate-wide protein orthogroups were defined using OrthoFinder version 2.5.4 (-S diamond -M msa -T raxml) [81] among proteomes of 17 species, involving 4 classes (Table 2). The program r8s verison 1.81 [82] was used to construct an ultrametric phylogenetic tree based on a rooted species tree (SpeciesTree_rooted.txt inferred by OrthoFinder). Then this ultrametric phylogenetic tree was utilized by CAFE (Computational Analysis of Gene Family Evolution) version 4.2.1 to identify significantly expanded/contracted gene families [83]. The divergence time of *Paramecium tetraurelia* and *Tetrahymena thermophila* (median time: 609.8 MYA) obtained through TimeTree (http://timetree.org/) was used as an input parameter for r8s to estimate the divergence time of all 17 ciliates.

**Table 2:** The macronuclear (MAC) genomes and amino acid sequences sources of 17 ciliates

Class	Order	Species	MAC genomes and amino acid sequence sources
Spirotrichea	Sporadotrichida	*Stylonychia lemnae*	http://ciliates.org/
		*Oxytricha trifallax*	http://ciliates.org/
		*Halteria grandinella*	https://www.ncbi.nlm.nih.gov/
	Urostylida	*Pseudokeronopsis flava*	https://www.ncbi.nlm.nih.gov/
		*Pseudokeronopsis carnea*	https://www.ncbi.nlm.nih.gov/
		*Pseudourostyla cristata*	present work
	Euplotida	*Euplotes octocarinatus*	http://ciliates.ihb.ac.cn/
		*Euplotes aediculatus*	http://ciliates.org/
		*Euplotes vannus*	http://ciliates.org/
Oligohymenophorea	Peniculida	*Paramecium tetraurelia*	https://www.ncbi.nlm.nih.gov/
		*Paramecium dodecaurelia*	https://www.ncbi.nlm.nih.gov/
	Tetrahymenida	*Tetrahymena thermophila*	http://ciliates.org/
		*Tetrahymena borealis*	http://ciliates.ihb.ac.cn/tcgd/
	Ophryoglenida	*Ichthyophthirius multifiliis*	http://ciliates.org/
	Philasterida	*Pseudocohnilembus persalinus*	http://ciliates.ihb.ac.cn/
Heterotrichea	Heterotrichida	*Stentor coeruleus*	http://ciliates.org/
Litostomatea	Entodiniomorphida	*Entodinium caudatum*	asked for data from the author [37]

The GO and KEGG pathway enrichment analyses were performed using the OmicShare tools (https://www.omicshare.com/tools) based on gene lists of expanded gene families, and also GO and KEGG annotation files of all genes.

### Analysis of chitin synthase

Searches for *CHS* genes of nine ciliates (*Blepharisma stoltei*, *Stentor coeruleus*, *Tetrahymena thermophila*, *Pseudocohnilembus persalinus*, *Paramecium tetraurelia*, *Pseudourostyla cristata*, *Oxytricha trifallax*, *Stylonychia lemnae*, and *Euplotes vannus*) and two fungi (*Aspergillus fumigatus* (GenBank assembly accession: GCA_000002655.1), *Saccharomyces cerevisiae* (GenBank assembly accession: GCA_000146045.2) were performed using HMMER [84] and essential gene model. This model was constructed based on the hidden Markov model profiles of the *CHS* gene family (Pfam: PF03142).

Multiple sequence alignments of CHS proteins were computed using the GUIDANCE2 server (http://guidance.tau.ac.il/ver2/) with the MAFFT algorithm. Based on alignment results, a consensus tree was constructed through IQ-TREE version 2.1.4 (-m MFP -B 1000 -bnni) with a maximum of 1,000 ultrafast bootstrap replicates and nearest-neighbor interchange optimization using models selected by MFP ModelFinder [85]. Motif analysis of protein sequence was conducted on the MEME website (http://meme-suite.org/tools/meme). Conserved domains were identified by NCBI Conserved Domain Search (CD-Search, http://www.ncbi.nlm.nih.gov/Structure/cdd/wrpsb.cgi). The information of the exon was extracted by annotation files. Finally, all the above information was aggregated in the TBtools software [75] and plotted. Additionally, the RNA-seq data of nine corresponding ciliates were collected from the NCBI SRA database (*B. stoltei* ERR6049484, *S. coeruleus* SRR5043309, *O. trifallax* SRR5027949, *S. lemnae* SRR2351389, *E. vannus* SRR7662949, *P. tetraurelia* SRR19666444, *T. thermophila* SRR17507285, *P. persalinus* SRR1768438). Then, RNA reads of all species were aligned to their reference genome respectively using HISAT2 version 2.1.10 [72] and normalized gene expression values were calculated by featureCounts version 2.0.1 [86] as transcripts per million (TPM).

### Identification of spliceosomal snRNAs and spliceosomal proteins in *P. cristata* and *O. trifallax*

According to Nuadthaisong et al. (2022) [56] with a slight modification, alignments of all U-small nuclear RNAs (snRNAs) from spliceosomes were downloaded from Rfam (U1, Rfam: RF00003; U2, Rfam: RF00004; U4, Rfam: RF00015; U5, Rfam: RF00020; U6, Rfam: RF00026; U11, Rfam: RF00548; U12, Rfam: RF00007; U4atac, Rfam: RF00618; U6atac, Rfam: RF00619) and used as the reference database. Then the spliceosomal snRNA genes of *P. cristata* and *O. trifallax* were identified by searching against the database using INFERNAL software (version 1.1.4).

To identify protein components of the spliceosome in *P. cristata* and *O. trifallax* genome, protein sequences of both species were searched against the Uniprot database using BLASTP (E-value cutoff = 1e-5). The hit results are searched again to the reference data in Nuadthaisong et al. (2022) [56] to determine corresponding spliceosomal proteins.

### Electronic supplementary material

Below is the link to the electronic supplementary material.

Supplementary Material 1

## Data Availability

The final genome assembly, genomic reads, and RNA-seq reads have been deposited in the CNSA (https://db.cngb.org/cnsa/) of CNGBdb (genome assembly: CNA0069573, genomic reads: CNX0767318, RNA-seq reads: CNX0767337). For genome annotation file, predicted coding and protein sequences, please email corresponding author.
