# Supplementary material for: Decryption of the survival “black box”: gene family expansion promotes the encystment in ciliated protists
Source: BMC Genomics. 2024 Mar 18;25:286. doi: 10.1186/s12864-024-10207-3 (PMC10946202; doi:10.1186/s12864-024-10207-3)
Supplement: Supplementary file 1 — Supplementary Material 1 [file 12864_2024_10207_MOESM1_ESM.doc]

**Table S1** Comparison of morphological features in six hypotrichous ciliates

| Species | Order | Average GC content | Genome size (Mb) | Body length (μm) | Habitat | Macronuclear number | Movement speed | Feeding habits | Cortical granule | Contractile vacuole |
| --- | --- | --- | --- | --- | --- | --- | --- | --- | --- | --- |
| *P. cristata* | Urostylida | 28% | 87 | 280-400 | freshwater/brackish water | dozens | slightly slow | bacteria, algae, smaller protists | + | + |
| *P. carnea* | Urostylida | 42% | 77 | 240~350 | sea | more than 100 | slow | protozoa (including Unicellular algae) | + | + |
| *P. flava* | Urostylida | 40% | 53 | 150~240 | sea/brackish water | dozens | slow | protozoa (including Unicellular algae) | + | + |
| *S. lemnae* | Sporadotrichida | 31% | 50 | 230 | freshwater | 2 | fast | omnivorous (bacteria, diatoms, dinoflagellates, chrysophytes, cryptophytes, and chlorophytes) | - | + |
| *O. trifallax* | Sporadotrichida | 32% | 106 | 100~160 | freshwater/brackish water | 2 | fast | omnivorous (bacteria, diatoms, dinoflagellates, chrysophytes, cryptophytes, and chlorophytes) | - | + |
| *H. grandinella* | Sporadotrichida | 43% | 63 | 24-36 | freshwater | 1 | fast | omnivorous (bacteria, diatoms, dinoflagellates, chrysophytes, cryptophytes, and chlorophytes) | - | + |


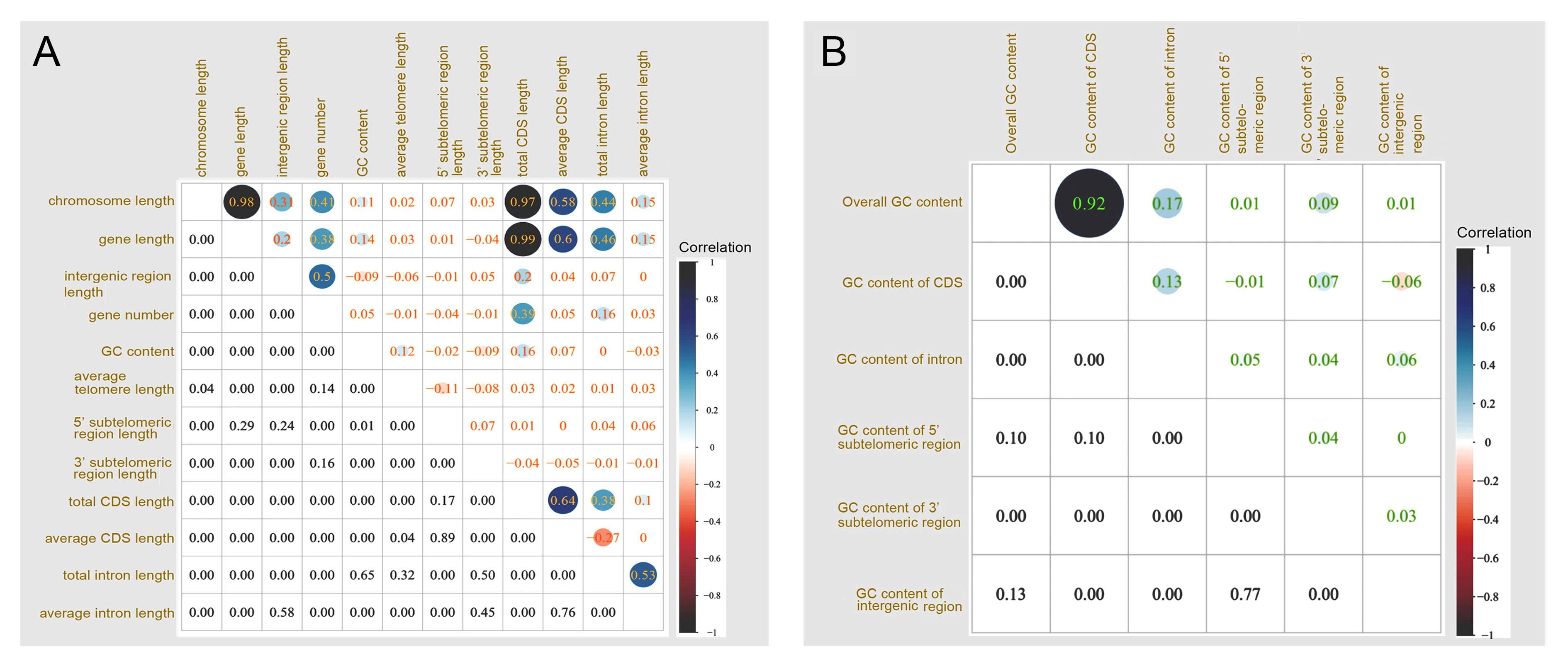
**Fig. S1** nanochromosome features in *Pseudourostyla cristata*. **A** Spearman correlation between nanochromosomal length with different nanochromosomal features of *P. cristata*. **B** Spearman correlation between overall GC content of nanochromosomes with GC content of different nanochromosomal structures of *P. cristata*. The circles and numbers in the upper triangle represent correlation coefficients between different genomic features. The numbers in the low triangle represent the *p*-value calculated with Spearman correlation analysis.

**Fig. S**
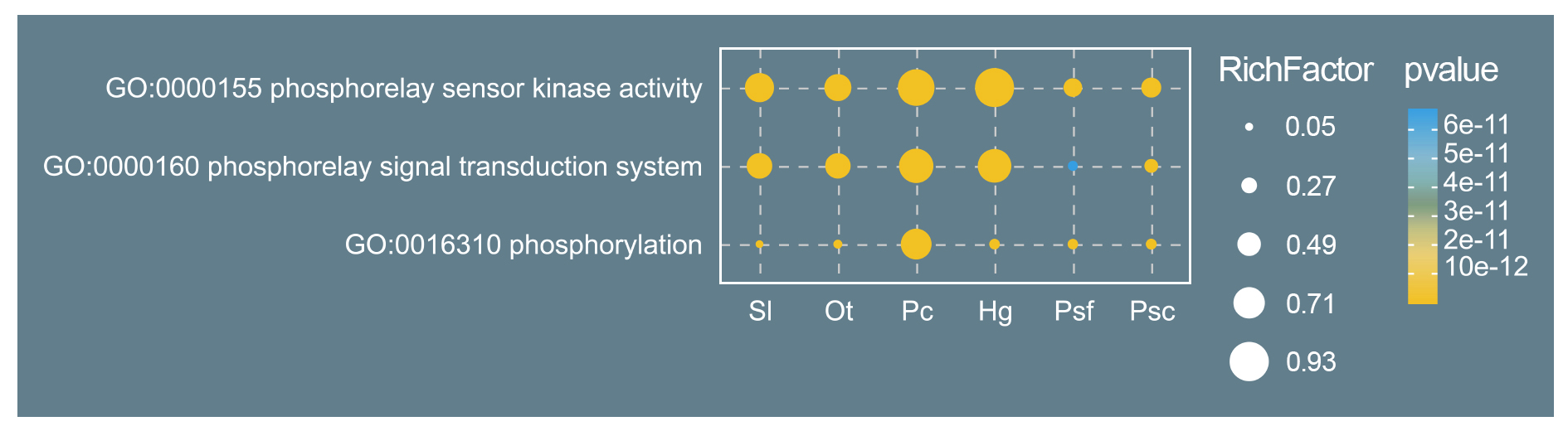
**2** Bubble plot showing enriched GO terms of the significantly expanded gene families with *p*-values less than 0.05 shared in six hypotrichs. Sl, *Stylonychia lemnae*; Ot, *Oxytricha trifallax*; Pc, *Pseudourostyla cristata*; Hg, *Halteria grandinella*; Psc, *Pseudokeronopsis carnea*; Psf, *Pseudokeronopsis flava*; GO, Gene Ontology. RichFactor = the ratio of enriched gene number to all gene number in this pathway term.


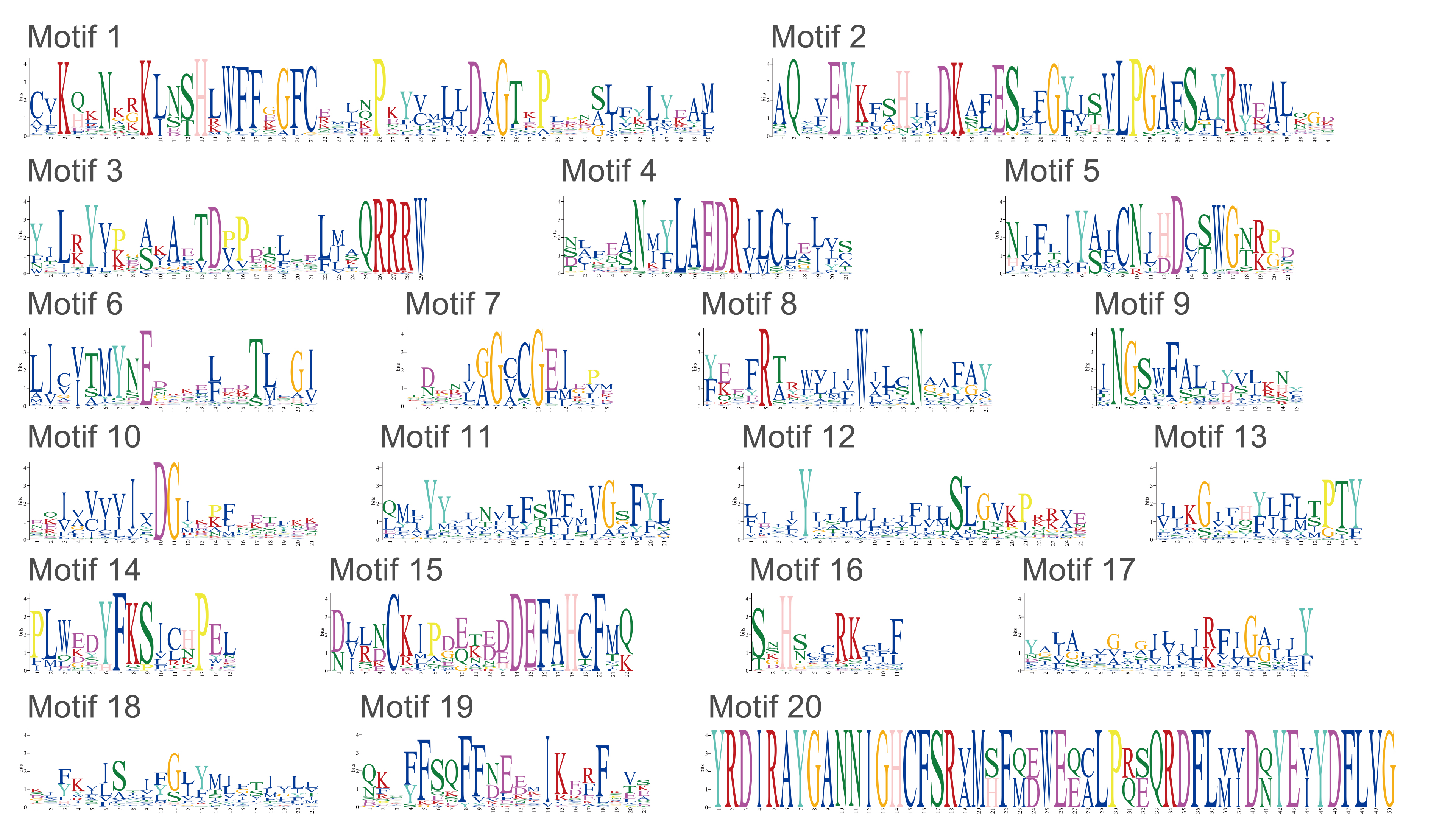
**Fig. S3** Motifs in chitin synthases proteins
